# Constitutional Mutations in *RTEL1* Cause Severe Dyskeratosis Congenita

**DOI:** 10.1016/j.ajhg.2013.02.001

**Published:** 2013-03-07

**Authors:** Amanda J. Walne, Tom Vulliamy, Michael Kirwan, Vincent Plagnol, Inderjeet Dokal

**Affiliations:** 1 Centre for Paediatrics, Barts and The London School of Medicine and Dentistry, Queen Mary University of London, Barts and The London Children’s Hospital, London E1 2AT, UK; 2 University College London Genetics Institute, Gower Street, London WC1E 6BT, UK

## Abstract

Dyskeratosis congenita (DC) and its phenotypically severe variant, Hoyeraal-Hreidarsson syndrome (HHS), are multisystem bone-marrow-failure syndromes in which the principal pathology is defective telomere maintenance. The genetic basis of many cases of DC and HHS remains unknown. Using whole-exome sequencing, we identified biallelic mutations in *RTEL1*, encoding a helicase essential for telomere maintenance and regulation of homologous recombination, in an individual with familial HHS. Additional screening of *RTEL1* identified biallelic mutations in 6/23 index cases with HHS but none in 102 DC or DC-like cases. All 11 mutations in ten HHS individuals from seven families segregated in an autosomal-recessive manner, and telomere lengths were significantly shorter in cases than in controls (p = 0.0003). This group had significantly higher levels of telomeric circles, produced as a consequence of incorrect processing of telomere ends, than did controls (p = 0.0148). These biallelic *RTEL1* mutations are responsible for a major subgroup (∼29%) of HHS. Our studies show that cells harboring these mutations have significant defects in telomere maintenance, but not in homologous recombination, and that incorrect resolution of T-loops is a mechanism for telomere shortening and disease causation in humans. They also demonstrate the severe multisystem consequences of its dysfunction.

## Main Text

Dyskeratosis congenita (DC [MIM 305000]) is a complex bone-marrow (BM)-failure syndrome in which the principal pathology is defective telomere maintenance and is associated with short telomeres.^1–3^ Eight of the genes known to be mutated in DC are involved in telomere maintenance and account for the genetic basis of approximately 60% of DC cases.^3–6^ The classical presentation of DC includes abnormal skin pigmentation, nail dystrophy, and oral leukoplakia. Individuals with DC frequently develop BM failure and are at a high risk of developing cancer, as well as a variety of other features.^7^ Hoyeraal-Hreidarsson syndrome (HHS [MIM 300240]) is a phenotypically severe DC variant that is characterized by a variety of features, including BM failure, intrauterine growth restriction, developmental delay, cerebellar hypoplasia, immunodeficiency,^8^ and usually short telomeres. Although the genetic basis of some cases of HHS has been elucidated, in many cases it remains unknown.

We recruited individuals who were classified as having DC on the basis of previously published clinical criteria^7^ to the Dyskeratosis Congenita Registry (DCR) in London. They were also included if they had at least four of six of the most commonly recognized HHS-associated features (intrauterine growth restriction, developmental delay, microcephaly, cerebellar hypoplasia, immunodeficiency, and BM failure). All samples were obtained with informed consent and the approval of our local ethics committee.

We performed whole-exome sequencing on an individual with genetically uncharacterized familial HHS by using the TruSeq Exome enrichment kit (Illumina, UK). The Illumina HighSeq 2000 system was used for generating 100 bp paired-end reads. Sequencing data were processed through the Illumina pipeline, and rare and very rare variants were identified by filtering against variations reported in dbSNP and the 1000 Genomes Project. It was hoped that selecting an individual with a severe phenotype would enrich a group of clinically matched index cases with highly penetrant disease-causing mutations. Under the assumption of an autosomal-recessive mode of inheritance, seven genes remained as possible candidates (Table S1, available online). On the basis of function, *RTEL1* (regulator of telomere length 1 [MIM 608833]) warranted further investigation. RTEL1 is an essential helicase that is crucial for telomere maintenance and DNA repair. In mice, it has been shown to play a critical role in genome stability because its knockout causes embryonic death, telomere loss, and chromosome fusions.^9^ RTEL1 has also been implicated as an antirecombinase in that it disrupts D-loop formation during homologous recombination^10^ and is essential for the disassembly of T-loops during DNA replication.^11^

**Figure 1:**
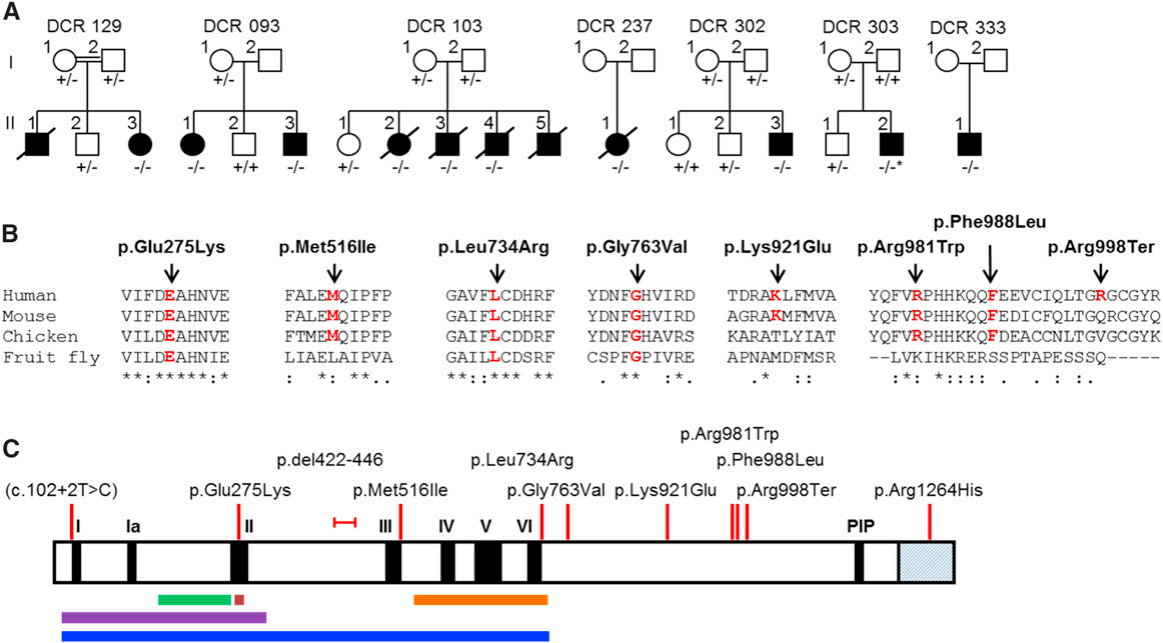
Segregation of *RTEL1* Mutations in HHS-Affected Families and the Location of RTEL1 Alterations in the Different Protein Domains (A) Segregation of *RTEL1* mutations causing HHS. Shown are seven families in which *RTEL1* mutations segregate as an autosomal-recessive trait. Where available, the genotype of each individual is shown as a plus sign for the wild-type allele and as a minus sign for the mutated allele. The asterisk indicates that the mutation appears to have arisen de novo. (B) Conservation of altered RTEL1 amino acids. Blocks of amino acid alignment were generated with ClustalW and show the degree of conservation of the altered amino acid residues in RTEL1. Sequences are as follows: human, *H. sapiens* (RTEL1 [RefSeq NP_116575.3]); mouse, *M. musculus* (RTEL1 [RefSeq NP_001001882.3]); chicken, *G. gallus* (RTEL1 [RefSeq XP_417435.3]); and fruit fly, *D. melanogaster* (RTEL1 [RefSeq NP_572254.1]). Asterisks indicate positions that have a single fully conserved residue, colons indicate conservation between groups of strongly similar properties, and periods indicate conservation between groups of weakly similar properties. (C) Conserved functional domains predicted in the RTEL1 amino acid sequence show the relative positions of the alterations caused by the mutations observed in our subject group. Domains are as follows: I–VI, helicase domains; PIP, proliferating cell nuclear antigen (PCNA)-interacting protein domain; green bar, iron-sulfur domain; purple bar, DEXDc2-DEAD-like helicase superfamily domain; brown bar, DEAH box; orange bar, helicase C-terminal domain; and blue bar, helicase, superfamily 1 and 2, ATP-binding domain, DinG/Rad3-type. The hatched C terminus is from isoform uc021wge.1.

Sanger sequencing of *RTEL1* (RefSeq accession number NM_032957.4) in the index case, her unaffected sibling, and her parents confirmed the presence and segregation of mutations c.2288G>T (p.Gly763Val) and c.3791G>A (p.Arg1264His) (this latter mutation is based on the alternative splice variant uc021wge.1 from the UCSC Genome Browser; DCR family 129 in Table 1 and Figure 1A) with disease. This suggests that these mutations could be causal in this family.

To determine whether *RTEL1* mutations are a significant cause of HHS, we extended the screen of *RTEL1* to a larger group of individuals. On the basis of the clinical definition of HHS and the presentation of the initial family, we selected 23 additional index cases with global BM failure and cerebellar hypoplasia from our DCR (Table S2). We scanned for mutations by denaturing high-performance liquid chromatography on a Transgenomic Wave DNA-fragment analysis system (Transgenomic, Glasgow, UK). Any fragments showing abnormal elution patterns were reamplified, and variants were confirmed by Sanger sequencing. We considered nonsynonymous, splice-site, or loss-of-function variants absent from the 1000 Genomes Project as potentially disease-causing mutations.

Biallelic *RTEL1* mutations were observed in a further six index cases, all of whom had a similar clinical phenotype, resulting in the identification of one homozygous and eight heterozygous mutations, of which six were missense, two affected the donor splice site, and one was a stop-gain mutation (Table 1, Figures 1B and 1C, and Table S3). The missense mutations identified occurred at residues that are generally highly conserved, and five of them aligned across mouse, chicken, and fruit fly proteins (Figure 1B). c.2941C>T (p.Arg981Trp) was recurrent—it was seen in three families (DCR families 237, 303, and 333). In total, 11 different *RTEL1* mutations were identified in ten cases from seven families, and all of them segregated with disease as an autosomal-recessive trait (Figure 1A).

To determine whether any of these variations had been described previously, we consulted the publically available data on the NHLBI Exome Sequencing Project Exome Variant Server (EVS). Of the mutations reported in this study, only one (c.1548G>T, reported at a frequency of 1/12,937 on the NHLBI EVS, accessed December 2012) had been reported on this website in over 12,000 exomes sequenced. We also had access to the exome data from an additional 60 individuals, and none of the variations reported here were seen in this group. Using the same filtering criteria (absent in the 1000 Genomes data set), we found the combined frequency of rare *RTEL1* nonsynonymous, loss-of-function, and splice variants in the NHLBI data set to be between 2.3% in European Americans and 3.4% in African Americans. Hence, the expected frequency of individuals carrying biallelic *RTEL1* variants was much lower in the control population (approximately 1/1,000) than in our disease group (6/23, chi-square p < 10^−10^). On the basis of this, we suggest that these mutations are causal for disease in a subset of individuals with HHS.

The biallelic mutations we identified are associated with a subset of HHS individuals with a tightly overlapping phenotype (Table 1). The key common features are global BM failure and cerebellar hypoplasia, and a large proportion also have microcephaly, intrauterine and extrauterine growth restriction, and immunodeficiency mainly affecting the B cell lineage. It is noteworthy that none of these individuals present with abnormal skin pigmentation, and less than half (∼41%) have any indications of nail dystrophy or leukoplakia. In contrast, the incidences of these three features in classical DC are approximately 89% (for abnormal skin pigmentation), 88% (for nail dystrophy), and 78% (for leukoplakia).^3^

Interestingly, c.1263+3A>G observed in DCR family 303 is a de novo mutation in this family’s index case. Bidirectional sequencing of both parents failed to identify this mutation. To examine the effect of this mutation, we extracted RNA from whole blood with a QIAGEN QIAamp RNA Blood Kit and generated cDNA by using reverse transcriptase (New England Biolabs, Hitchin, UK) and an oligo dT primer. Sanger sequencing of exons 13–17 demonstrated that c.1263+3A>G results in the skipping of exon 15 and thus causes an in-frame deletion of 24 amino acids (422–446, Figure S1).

To determine whether *RTEL1* mutations are specific to individuals with an HHS phenotype, we screened 102 index cases with DC or related BM-failure syndromes. No biallelic mutations were found in this large group, suggesting that biallelic *RTEL1* mutations are a relatively common cause of HHS, defined by presentation with a minimum of BM failure and cerebellar hypoplasia, but are not a major cause of DC or related BM-failure syndromes.

**Figure 2:**
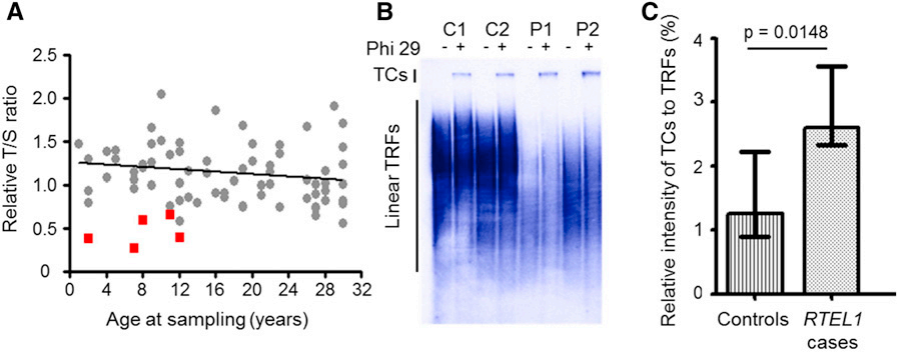
Functional Effects of *RTEL1* Mutations in Individuals with HHS (A) Telomere lengths measured by monochrome multiplex quantitative PCR are expressed as a telomere-to-single-copy-gene (T/S) ratio (individuals with biallelic *RTEL1* mutations are compared to controls). Red squares represent cases, and gray circles represent controls. (B) Mutations in *RTEL1* affect T-circle accumulation. Linear terminal restriction fragments (TRFs) and Phi-29-dependant T-circles (TCs) were detected in genomic DNA by Southern blot analysis. (C) Graphical representation of the increase in the production of T-circles in cases (P1 and P2) and controls (C1–C3). A box plot of the median value from three different experiments shows the interquartile range (p = 0.0148, unpaired t test).

Telomeres are complex nucleoprotein structures that protect chromosome ends from cellular exonucleases and nonhomologous-end joining and distinguish them from double-stranded breaks, which can occur elsewhere in the genome. This protection is achieved not only by the involvement of proteins of the shelterin complex^13^ but also by the creation of a lasso-like configuration called a T-loop by the 3′ end of telomeres.^14^ In this structure, a 3′ single-stranded overhang invades the double-stranded telomeric region and displaces the identical strand of DNA to form a displacement-loop (D-loop) at the base of the T-loop. For DNA replication to occur, these loops need to be resolved while maintaining telomere integrity. It is also important that these telomeric-strand invasions are distinguished from the preliminary step of a homologous recombination event during double-strand break repair.^13^ Conditional mouse knockout studies have demonstrated that RTEL1 is essential for telomere maintenance in terms of both length and stability.^10^ To determine whether this observation holds true in individuals with *RTEL1* mutations, we used quantitative PCR to measure telomere length in samples for which we had DNA of sufficient quality (n = 5). A monochrome multiplex quantitative PCR method adapted for use on a LightCycler 480 real-time thermocycler (Roche, Little Chalfont, UK) was used as described previously.^15,16^ The telomere lengths were significantly shorter in the group with biallelic *RTEL1* mutations than in controls (n = 71) (p = 0.0003, Mann-Whitney U, Figure 2A).

In mice, RTEL1 is essential for survival—it is required during telomere replication, in DNA-damage repair (specifically in homologous recombination), and for efficient elongation of the telomere by telomerase.^17^ Vannier et al. demonstrated that RTEL1 is necessary to cause T-loop disassembly and that T-loops only form with 3′ single-stranded DNA overhangs.^11^ It has been suggested that persistent T-loops are inappropriately resolved by the SLX4 complex, which cleaves the T-loop from the telomere. This results in the formation of T-circles and a corresponding loss in telomere length.^13,17,18^ When RTEL1 is inactivated, T-circle formation increases. To examine this potential mode of telomere shortening in individuals with biallelic *RTEL1* mutations, we utilized a T-circle amplification assay.^19^ The formation of T-circles in genomic DNA from controls and the index cases from DCR families 302 and 303 (for whom there was good-quality genomic DNA) was measured (Figure 2B). Compared to the signal from the telomeric terminal restriction fragment, the relative signal from the T-circles was increased in the index cases (p = 0.0148, unpaired t test, Figure 2C), suggesting that these mutations affect the ability of RTEL1 to correctly process T-loops and thus result in telomere shortening and a relative increase in T-circle formation. By contrast, there was no significant difference in T-circle intensity between five HHS cases with *DKC1* (MIM 300126) mutations and the normal controls (p = 0.54, Mann Whitney U, Figure S2). These data suggest that T-circle production is increased in individuals with biallelic *RTEL1* mutations, which is consistent with observation in mice and supports a disease-causing mechanism that results in the shortening of telomeres without impacting the function of the telomerase complex.

The alterations caused by the mutations identified in this study were spread throughout RTEL1 (Figure 1C), and four were within the RAD3-related helicase domain. Helicases are a group of proteins that function by using ATP hydrolysis to catalyze the unwinding of polynucleic-acid structures and are involved in DNA repair, replication, recombination, chromosomal segregation, and telomere maintenance.^20^ They can be classified into several families, some of which are associated with human disease. One such family is the RECQ helicase family; alterations in WRN, BLM, and RECQL4 cause Werner syndrome (MIM 604611), Bloom syndrome (MIM 604610), and Rothmund-Thomson syndrome (MIM 603780),^21^ respectively. The most recently identified helicase family with telomere effects is the FANCJ family, which has several members, including BRIP1 (FANCJ) and RTEL1. BRIP1 is associated with Fanconi anemia (FA [MIM 227650]),^22^ a disorder characterized by DNA repair and/or homologous recombination defects, and RTEL1 (shown in this study) is associated with HHS. These diseases have some overlapping features, but there are also clear differences. None of the RECQ-associated diseases have global BM failure, but there is an increased risk of cancers, whereas both FA and DC are characterized by BM failure. Peripheral-blood lymphocytes from several individuals with biallelic *RTEL1* mutations were tested for chromosomal breakage after exposure to DNA cross-linking agents in the standard FA test (Table 1), but unlike lymphocytes from individuals with FA, they had no increased chromosomal breakage. This demonstrates that lymphocytes with biallelic *RTEL1* mutations do not exhibit the same cellular defects as lymphocytes from individuals with FA, suggesting that cells with biallelic *RTEL1* mutations do not have significant defects in DNA repair and/or homologous recombination.

Two studies have previously suggested that HHS might arise as a result of defects in telomere biology other than just length. Touzot et al.^23^ showed that individuals with HHS can have a variety of telomere defects. Critically short telomeres are not inevitably associated with this severe phenotype, and additional factors, some of which are involved in telomere protection and/or replication, might cause some cases of HHS. Lamm et al.^12^ presented findings on a family reported here (DCR family 103) and noted that leukocyte telomeres were severely short (as observed in our study) but that fibroblast telomeres were of normal length. They also found that the 3′ single-stranded overhang was reduced in both cell types and that the catalytic telomerase core functioned normally. Mutations identified in *RTEL1* might now explain some of their observations.

In summary, we have identified biallelic *RTEL1* mutations responsible for a subgroup of HHS, a severe variant of DC. We have also demonstrated that defective human RTEL1 has a detrimental effect on telomere maintenance, suggesting that incorrect resolution of T-loops is a mechanism for telomere shortening in humans. Although previous studies have suggested that RTEL1 is an essential helicase for both telomere maintenance and the regulation of homologous recombination, our study shows that cells harboring biallelic *RTEL1* mutations only have significant defects in telomere maintenance. The identification of mutations in *RTEL1* extends the sphere of diseases that can be classified as “telomereopathies” and adds to the number of diseases that are caused by defective helicases.

## Figures and Tables

**Table 1:** Common Features of Individuals with Biallelic *RTEL1* Mutations

	**Families from the Dyskeratosis Congenita Registry**											
	**129**		**093**		**103**^a^				**237**	**302**	**303**	**333**
	**II-1**	**II-3**	**II-1**	**II-3**	**II-2**	**II-3**	**II-4**	**II-5**	**II-1**	**II-3**	**II-2**	**II-1**
Mutation(s)	NA	c.2288G>T (p.Gly763Val)	c.2964T>G (p.Phe988Leu) (homozygous)		c.1548G>T (p.Met516Ile)			NA	c.102+2T>C (splice site)^c^	c.2201T>G (p.Leu734Arg)	c.1263+3A>G (p.del422_446)	c.823G>A (p.Glu275Lys)
		c.3791G>A (p.Arg1264His)^b^			c.2992C>T (p.Arg998Ter)				c.2941C>T (p.Arg981Trp)	c.2761A>G (p.Lys921Glu)	c.2941C>T (p.Arg981Trp)	c.2941C>T (p.Arg981Trp)
Gender	male	female	female	male	female	male	female	male	female	female	male	male
Age at report or (death) in years	(3)	8	16	(9)	16	(4)	(11)	(4)	(4)	2	12	6
Global bone-marrow failure	+	+	+	+	+	+	+	+	+	+	+	+
Immunodeficiency	+	+	−	unknown	+	+	+	+	+	+	+/−	−
Cerebellar hypoplasia	+	−	−	+	+	+	+	+	+	+	+	+
Microcephaly	+	−	−	+	+	+	+	+	+	+	+	−
IUGR	+	−	−	+	+	+	+	+	+	+	+	−
Growth retardation	+	−	−	+	−	+	+	+	+	+	+	+
Developmental delay	+	−	−	+	−	+	+	+	−	+	+	+
Abnormal skin pigmentation	−	−	−	−	−	−	−	−	−	−	−	−
Nail dystrophy	−	−	+/−	−	+	−	+	−	−	+	+	−
Leukoplakia	−	−	+	−	+/−	−	+	−	−	+	−	+
Other features	−	−	−	hypogonadism	−	−	−	−	−	esophageal web and enteropathy	dysphagia	abnormal facies and intracranial calcification
Increased chromosomal breakage with DEB and/or MMC	−	−	unknown	−	−	−	−	−	−	−	unknown	−

The following abbreviations are used: IUGR, intrauterine growth restriction; DEB, diepoxybutane; MMC, mitomycin C; +, present feature; –, absent feature; +/−, some suggestion that feature is present; and NA, sample not available for analysis.

a Family previously published.^12^

b Mutation and predicted protein change were called from the alternative splice variant uc021wge.1.

c Predicted to affect splicing.

## Web Resources

The URLs for data presented herein are as follows:

1000 Genomes, http://browser.1000genomes.org
ClustalW2 - Multiple Sequence Alignment, http://www.ebi.ac.uk/Tools/msa/clustalw2/
dbSNP, http://www.ncbi.nlm.nih.gov/projects/SNP/
NHLBI Exome Variant Server Exome Sequencing Project (ESP), http://evs.gs.washington.edu/EVS/
Online Mendelian Inheritance in Man (OMIM), http://www.omim.org/
RefSeq, http://www.ncbi.nlm.nih.gov/RefSeq
UCSC Human Genome Browser, http://genome.ucsc.edu/cgi-bin/hgGateway

## Note Added in Proof

While this manuscript was under review, Ballew et al. published a paper entitled “Germline mutations of regulator of telomere elongation helicase 1, RTEL1, in dyskeratosis congenita” (Hum. Genet., published online January 18, 2013). This paper documents one family affected by biallelic *RTEL1* mutations causing HHS and two families affected by heterozygous *RTEL1* mutations (causing HHS in one and causing DC in the other). Although the family afflicted with biallelic *RTEL1* mutations is similar to the seven families described in this study, the pathogenic role of the heterozygous *RTEL1* mutations in the other two families is unclear.
