# Supplementary material for: Constitutional Mutations in RTEL1 Cause Severe Dyskeratosis Congenita
Source: Am J Hum Genet. 2013 Mar 7;92(3):448–53. doi: 10.1016/j.ajhg.2013.02.001 (PMC3591859; doi:10.1016/j.ajhg.2013.02.001)
Supplement: Document S1. Figures S1 and S2 and Tables S1–S3 [file mmc1.pdf]

The American Journal of Human Genetics, Volume *92*

Supplemental Data

Constitutional Mutations in *RTEL1*

Cause Severe Dyskeratosis Congenita

Amanda J. Walne, Tom Vulliamy, Michael Kirwan, Vincent Plagnol, and Inderjeet Dokal

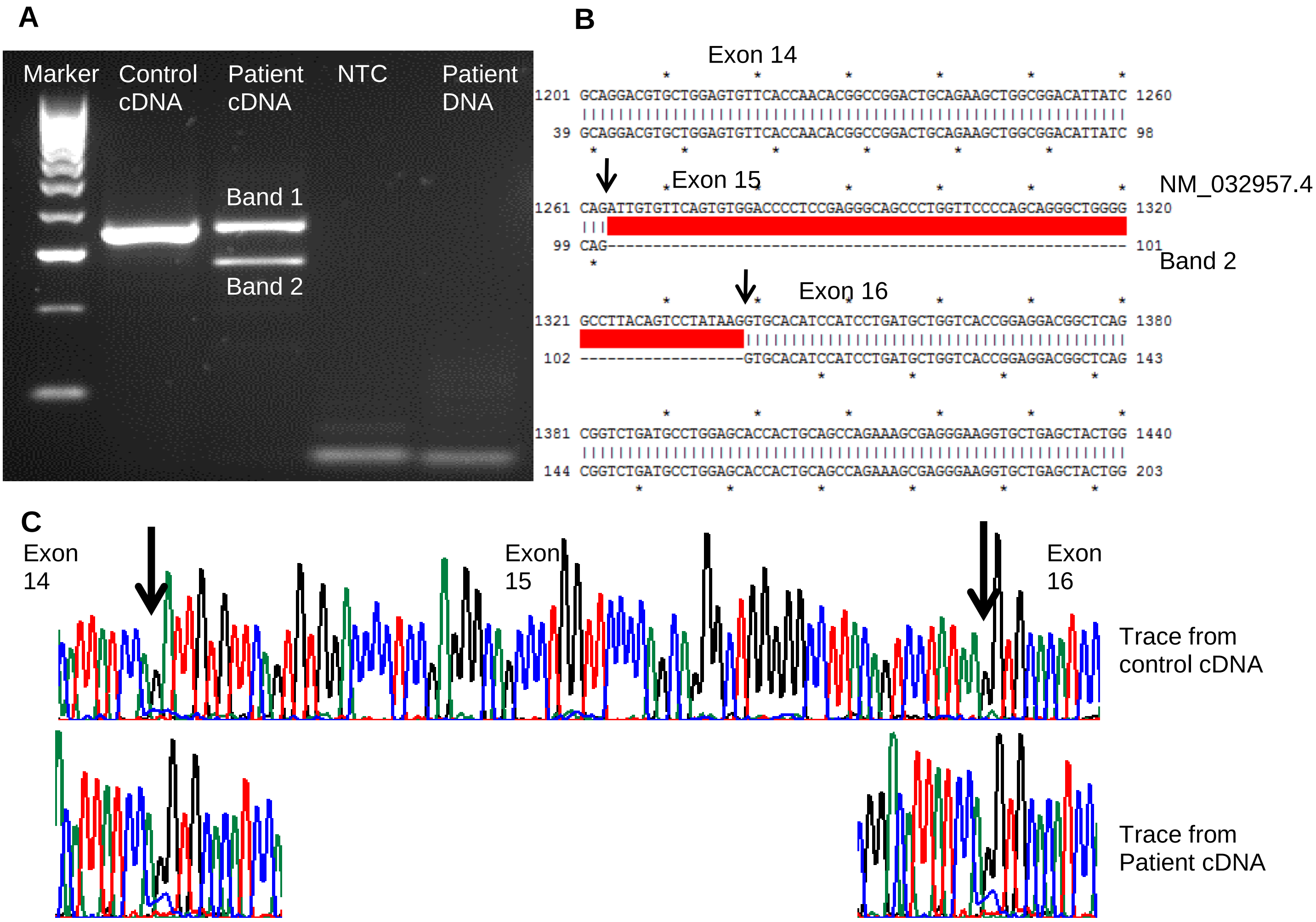

Figure S1. Confirmation that the c.1263+3A>G Mutation Disrupts Splicing

(A) Gel image showing the presence of the splice variant caused by the genomic mutation c.1263+3A>G. Band 2 was excised from the gel and sequenced. NTC = no template control. Patient DNA was included to show the primers gave a product that was unique to the cDNA.

(B) Sequence alignment of the reference with Band 2 showing that exon 15 is spliced from the variant in frame.

(C) Control sequencing trace compared with Band 2 trace. Arrows show exon boundaries.

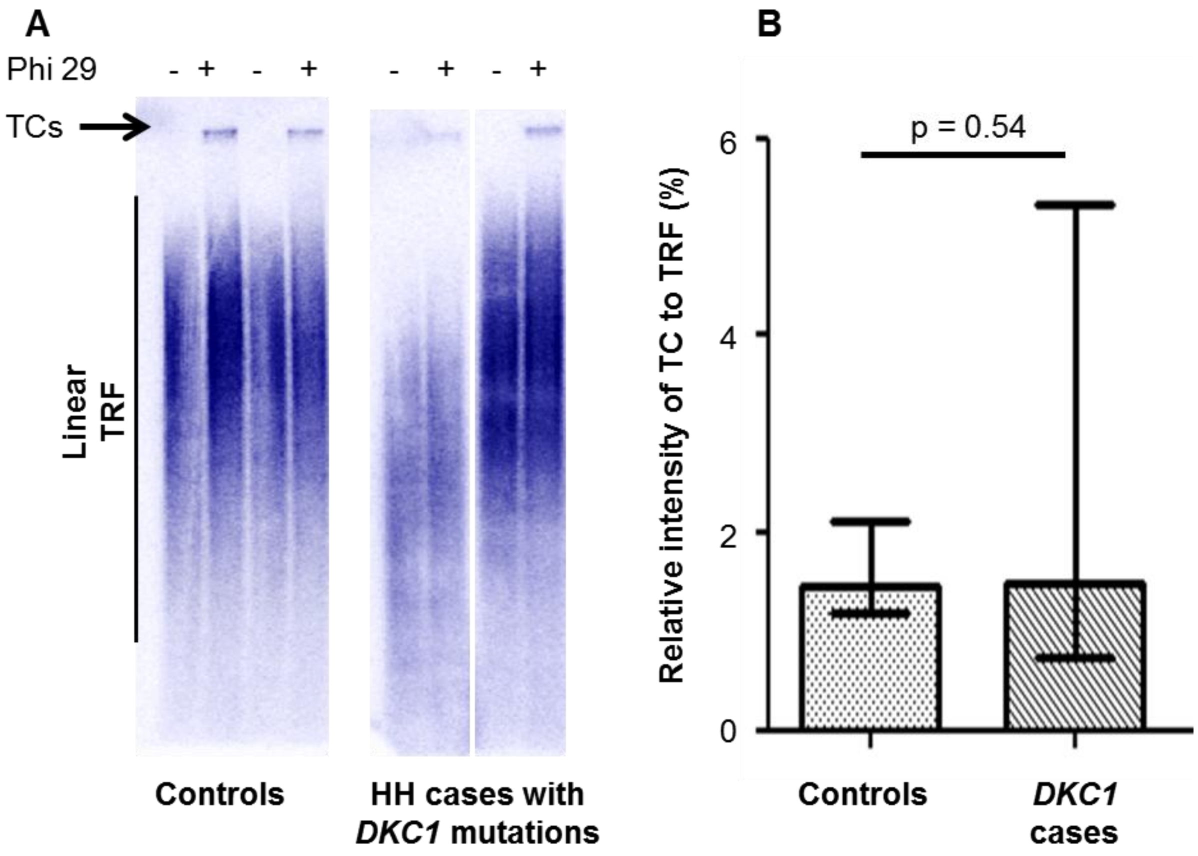

Figure S2. T-Circle Production in HH Cases with *DKC1* Mutations Is Not Increased Compared with Controls

(A) Mutations in *DKC1* have no significant effect on T-circle accumulation. Linear terminal restriction fragments (TRF) and Phi-29 dependant T-circles (TCs) are detected in genomic DNA by Southern blot analysis.

(B) Graphical representation of the intensity of T-circles to TRF in controls (n=10) and HH cases with *DKC1* mutations (n=5). Box plot of median value also showing interquartile range, p value calculated using a Mann Whitney U test.

Table S1. List of Potential Candidate Genes Remaining after Exome Sequencing of DCR129

Gene	Full Name	MIM Number	AA Change 1	AA Change 2	Chr.	Function
BBX	bobby sox homolog (Drosophila)	N/A	p.A139T	p.S22N	3	Transcription factor
FAM38A	family with sequence similarity 38, member A	611184	p.Q1036delinsQER	p.Q1032delinsQEE	16	Ion channel component
ANKLE1	ankyrin repeat and LEM domain containing 1	N/A	p.643_644del	p.643_644del	19	Induced DNA cleavage and damage response
WDR87	WD repeat domain 87	N/A	p.2295_2301del	p.2295_2301del	19	Also known as testes development protein
ZNF714	zinc finger protein 714	N/A	p.Q69X	p.Y102delinsYN	19	Associated with cleft lip syndrome
RTEL1	regulator of telomere elongation helicase 1	608833	p.Gly763Val	p.Arg1264His	20	Telomere elongation and stability
NEFH	neurofilament, heavy polypeptide	162230	p.E650delinsEQAKSPE	p.K647delinsRSPEKAK	22	Biomarker of neuronal damage

Table S2. Clinical Features of Individuals with HH Screened in This Study Who Did Not Have Biallelic *RTEL1* Mutations

**DCR/DCV**	**80**	**99**	**102**	**118**	**144**	**159**	**162**	**179**	**223**	**259**	**291**	**309**	**312**	**326**	**361**	**373**	**DCV206**
Age at report	<1	2	2	<1	13	3	5	2	2	1	2	1	3	8	0	10	22
Sex	M	F	M	M	?	M	M	F	F	M	F	M	M	M	M	M	M
Global bone marrow failure	yes	yes	yes	yes	yes	yes	yes	yes	yes	yes	yes	yes	yes	yes	yes	yes	yes
Immuno-deficiency			yes		yes			yes	yes				yes		yes		
Cerebellar Hypoplasia	yes	yes	yes	yes	yes	yes	yes	yes	yes	yes	yes	yes	yes	yes	yes	yes	yes
Microcephaly			yes	yes	yes	yes		yes	yes	yes	yes			yes		yes	yes
IUGR				yes		yes		yes	yes			yes			yes		
Growth retardation	yes		yes	yes		yes					yes			yes			yes
Developmental delay	yes	yes	yes	yes	yes	yes	yes	yes	yes	yes				yes		yes	yes
Abnormal skin pigmentation						yes					yes					yes	
Nail dystrophy		yes				yes					yes			yes			
Leukoplakia		yes	yes			yes					yes						

IUGR = Intrauterine growth restriction; DCV = DC variant, this represents a group of patients who have a bone marrow failure disorder but not enough clinical features to be entered into the DCR.

Table S3. Location and Predicted Consequence of Mutations Identified in *RTEL1*

Nucleotide Change[a]	Protein Change[a]	Exon	Protein Domain and/or Consequence	PolyPhen Score[b]	Zygosity
c.102+2T>C		2	Predicted to affect splicing	NA	Heterozygous
c.823G>A	p.Glu275Lys	9	DEAH box	1.00	Heterozygous
c.1263+3A>G	p.del422-446	15	skip exon 15[c]	NA	Heterozygous
c.1548 G>T	p.Met516Ile	17	dinG domain[d]	0.734	Heterozygous
c.2201 T>G	p.Leu734Arg	24	dinG domain	1.00	Heterozygous
c.2288 G>T	p.Gly763Val	25	dinG domain	1.00	Heterozygous
c.2761 A>G	p.Lys921Glu	29	Function unknown	0.971	Heterozygous
c.2941 C>T[e]	p.Arg981Trp[e]	30	Function unknown	1.00	Heterozygous
c.2964 T>G	p.Phe988Leu	30	Function unknown	0.486	Homozygous
c.2992 C>T	p.Arg998Ter	30	Premature truncation at amino acid 998	NA	Heterozygous
c.3791 G>A[f]	p.Arg1264His[f]	35	Function unknown	0.994	Heterozygous

[a] = numbering relative to NM_032.957.1 and NP_116575.3 except for [f]; [b] = polymorphism phenotyping; [c] = experimentally verified; [d] dinG domain = Rad3-related helicase domain; [e] = recurrent mutation; [f] = numbering relative to coding sequence and protein translation from isoform uc021wge.1; NA = not applicable.
